# Primary and secondary aortopathy associated with adult congenital heart disease - retrospective study

**DOI:** 10.1186/s13019-020-01283-z

**Published:** 2020-09-10

**Authors:** Ingrid Schusterova, Alžbeta Banovcinova, Marianna Vachalcova, Marta Jakubova, Panagiotis Artemiou

**Affiliations:** 1 1th Department of Cardiology, East Slovak Institute of Cardiovascular Diseases, Faculty of Medicine, Pavol Jozef Safarik University in Kosice, Kosice, Slovakia; 2 Department of Paediatrics and Adolescent Medicine, Faculty of Medicine, Pavol Jozef Safarik University in Kosice, Kosice, Slovakia; 3 Medical Faculty of the Comenius University, National Institute of Cardiovascular Diseases, Clinic of Cardiac Surgery, Pod krasnou horkou 1, 83101 Bratislava, Slovakia

**Keywords:** Primary/secondary aortopathy, Aortic root, Ascending aorta, Congenital heart disease

## Abstract

**Background:**

Primary and secondary aortopathy are frequently encountered in patients with congenital heart disease. The aim of this study is to present our experience and the incidence of primary and secondary adult CHD-associated aortopathy.

**Methods:**

The cohort is comprised of adult patients with congenital heart disease from the registry of the Eastern Slovakia Institute of Cardiovascular Diseases. Data from the last follow-up examinations are included in this study. In the primary and secondary aortopathy groups were 35 and 12 patients respectively. As a control group were selected 64 patients with non aortopathy associated congenital heart disease (atrial and ventricular septal defect).

**Results:**

Patients with primary and secondary aortopathy had larger ascending aorta/aortic root diameters than the control group (36.28 (26–49) mm vs 30.25 (21–41) mm *p* = 0.000113, 33.82 27–49) mm vs 29.03 (19–38)mm *p* = 0.000366 and 42.1 (30–50) mm vs 30.25 (21–41) mm, *p* = 0.000106, 35.67 (27–48) mm vs 29.03 (19–38) mm, *p* = 0.000119 respectively). Moreover, patients with secondary aortopathy had statistically significant larger ascending aorta diameter compared to the patients with primary aortopathy (42.1 (30–50) mm vs 36.28 (26–49) mm *p* = 0.030). During the follow-up period, were performed only in 2 patients (one from each group) operations on the aortic root and the ascending aorta due to aortic root or ascending aorta dilatation.

**Conclusion:**

More patients with secondary aortopathy had dilated ascending aorta/ aortic root, as well as larger aortic diameters compare to the patients with primary aortopathy. Routine follow-up of these patients with attention to aortic diameter is necessary.

## Background

Dilatation of the aortic root and the ascending aorta is frequently encountered in patients with congenital heart disease (CHD) at initial presentation and during follow-up.

Primary aortic dilatation is mainly associated with coarctation of the aorta (CoA), bicuspid aortic valve (BAV) and conotruncal abnormalities such as tetralogy of Fallot (TOF), pulmonary atresia with ventricular septal defect (PA/VSD) or truncus arteriosus (TAC). The evolution of the aortic size after birth will result from a combination of intrinsic pathology, hemodynamic factors, associated malformations, surgical or catheter interventions, and control of risk factors later in life [1].

Secondary dilatation of the aortic root and to a lesser extent of the ascending aorta, is seen after congenital cardiac surgery, when the original aortic root is replaced by a pulmonary autograft, as in the Ross procedure, or modified as in the arterial switch operation (ASO) or in systemic outflow tract reconstruction in single ventricle (SV) patients. In these situations the neo-aortic root consists mainly of pulmonary arterial root tissue introduced in the high pressure left-sided system, often leading to dilatation in a time-dependent fashion [1].

The dilatation of the aorta or the neo-aortic root is not a stand-alone characteristic, but needs to be regarded as a part of the aorto-ventricular complex, compromising the systemic ventricle, the aortic valve, the aortic root, and the vascular wall. Each component of this complex may by itself influence the other components, thus introducing a dysfunction at multiple levels, often defined as aortopathy [2].

CHD-associated aortopathy shows histological and functional similarities like Marfan syndrome, such as degeneration of the aortic media (cystic medial necrosis) [3, 4].

The aim of this study is to present our experience and the incidence of primary and secondary adult CHD-associated aortopathy.

## Methods

### Patients and methods

This is a retrospective study and the cohort is comprised of adult patients with congenital heart disease from the registry of the Eastern Slovakia Institute of Cardiovascular Diseases and all the relevant data were obtained from the medical records. Patients with CHD at the age of 18 years old are included in the adult CHD registry of the Eastern Slovakia Institute of Cardiovascular Diseases. In total 629 patients with CHD are enrolled in this registry. The mean follow-up duration of the entire cohort is 18,15 years. Regular annual follow-up examination with a transthoracic echocardiography (TTE) of the ascending aorta and the aortic root was made in 127 patients. Data from the last follow-up examinations which were done during the last year are included in this study. The primary and secondary aortopathy groups consisted of 47 patients in total, including 2 with CoA, 26 with BAV, 7 with TOF - (primary aortopathy group, 35 patients, which is 27,56% of all regularly followed patients), 4 after a Ross procedure, 6 after an arterial switch operation due to transposition of the great arteries (TGA) and 2 after a Fontan procedure - (secondary aortopathy group, 12 patients, which is 9,45% of all regularly followed patients). The control group was consisted of 64 patients with non aortopathy associated CHD (atrial and ventricular septal defect). The diameter of the aortic root and ascending aorta were measured by TTE and all the evaluations were done according to the standard techniques recommended by the American Society of Echocardiography [5]. The criteria for intervention on the aortic root and ascending aorta were according to the international guidelines and recommendations [3].

An informed consent from the patients and approval from the institutional review board were obtained in order to present this study.

### Statistical analysis

All variables were expressed as median and the qualitative variables as numbers and percentages. A one-way ANOVA and the Tukey’s range test paired were used to compare the variables. A *p* value of less than 0.05 s was considered statistically significant. The statistical analyses were performed using the StatSoft, Inc. (2007). STATISTICA (data analysis software system), version 8.0 www.statsoft.com

## Results

The median age of the patients in the primary and secondary aortopathy and the control groups were 30.65 (18–60), 29.24 (18–44) and 36.35 (19–66) years old respectively (Table 1).

**Table 1:** Characteristics of the study groups

	Median age (years)	Median ascending aorta (mm)	Median aortic root (mm)
Primary aortopathy	30.65	36.28	33.82
Secondary aortopathy	29.24	42.10	35.65
Control group	36.35	30.25	29.03

**Fig. 1:**
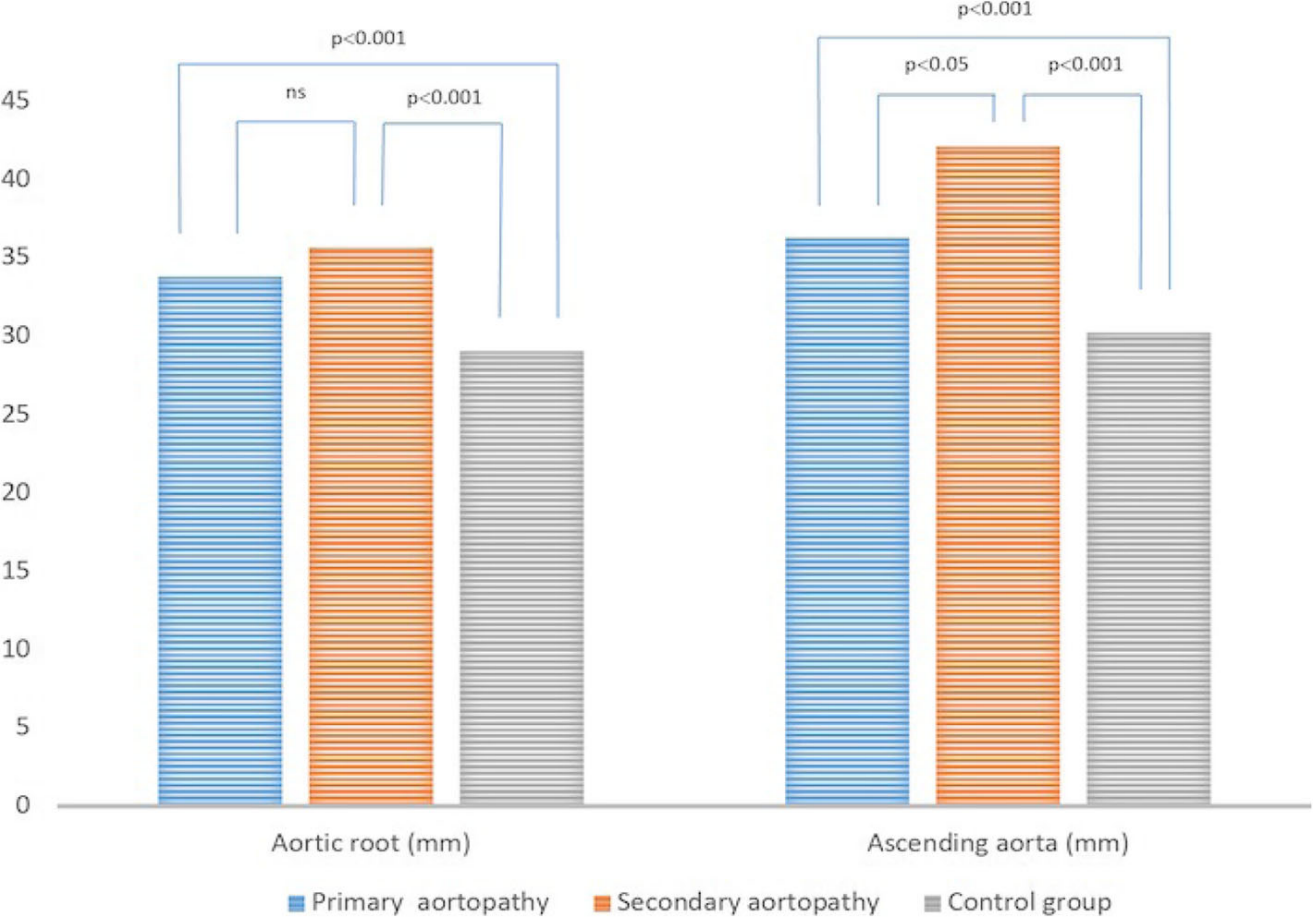
Aortic root and ascending aorta in the study groups- primary and secondary aortopathy and control groups

Patients with primary and secondary aortopathy had statistically significant larger aortic root diameter compared to the control group (35.67 (27–48) mm vs 29.03 (19–38) mm, *p* < 0.001), and (33.82 (27–49) mm vs 29.03 (19–38) mm, p < 0.001) respectively (Fig. 1, Table 1).

Moreover, patients with secondary aortopathy had also larger aortic root diameter than the patients with primary aortopathy (35.67 (27–48) mm vs 33.82 (27–49) mm *p* = 0.07), but with no statistical significance (Fig. 1, Table 1).

Patients with secondary aortopathy had statistically significant larger ascending aorta diameter compared to the control group (42.10 (30–50) mm vs 30.25 (21–41) mm, *p* < 0.001) as well as to the patients with primary aortopathy (42.10 (30–50) mm vs 36.28 (26–49) mm, *p* < 0.05) (Fig. 1, Table 1). Also, patients with primary aortopathy had statistically significant larger ascending aorta diameter compared to the control group (36.28 (26–49) mm vs 30.25 (21–41) mm, p < 0.001) (Fig. 1, Table 1).

From the primary aortopathy group, 8 patients (22.85%) had ascending aorta diameter between 35 and 40 mm and 9 patients (25.71%) > 40 mm, 7 patients (20.00%) had aortic root diameter between 35 and 40 mm and 6 patients (17.14%) > 40 mm (Table 2).

**Table 2:** Characteristics of aortic root and ascending aorta diameters in the study groups- primary and secondary aortopathy

	Ascending aorta 35–40 mm	Ascending aorta > 40 mm	Aortic root 35–40 mm	Aortic root > 40 mm
Primary aortopathy	8 (22.85%)	9 (25.71%)	7 (20.00%)	6 (17.14%)
Secondary aortopathy	3 (25.00%)	6 (50.00%)	2 (16.67%)	4 (33.33%)

From the secondary aortopathy group, 3 patients (25.00%) had ascending aorta diameter between 35 and 40 mm, and 6 patients (50.00%) > 40 mm, 2 patients (16.67%) had aortic root diameter between 35 and 40 mm and 4 patients (33.33%) > 40 mm (Table 2).

Nine patients (25.71%) from the primary aortopathy group had undergone at least one prior operation. One patient had a repair of CoA and later a reoperation due to subvalvular aortic stenosis, 1 patient with BAV had aortic valve replacement (AVR) and later a redo-AVR, 1 patient with BAV had replacement of the ascending aorta due to aortic aneurysm, and 6 patients had repair of TOF, where 3 of them had multiple reoperations.

All the patients in the secondary aortopathy group had undergone at least one prior operation. Four patients underwent a Ross procedure, where one of them later he had reoperation and replacement of the neo-aorta due to dilatation of the pulmonary homograft, 6 patients underwent switch operation due to TGA, and 2 patients had a Fontan procedure.

During the follow-up period, only 2 patients (one from each group) were operated due to aortic root or ascending aorta dilatation. The rest of the patients are under follow-up screening and so far they do not meet the indications for any procedure on the aortic root and the ascending aorta.

## Discussion

Progressive proximal aortic (aortic root and ascending aorta) dilatation is frequently found in adults with unrepaired (primary aortopathy) or repaired (secondary aortopathy) CHD. In our study, in the primary aortopathy group 25.71 and 17.14% of the patients had dilated ascending aorta and aortic root respectively. Similar results are reported also by other authors. Steward et al. [6] found dilated aortic root in 16%. There was a trend to a more abnormally widened aorta in patients who had surgery for repair of CoA later in life. Five of the patients in their series with a history of hypertension that died from aortic aneurysm rupture, had the original surgery at the mean age of 19 years old. It is unclear if early repair of CoA will always be able to prevent late aortic dilatation. Even neonatal intervention does not prevent the occurrence of late hypertension, which by itself may trigger aortic dilatation [7]. Biopsies studies of the aortic wall found increased amount of collagen and a decreased smooth muscle content in the pre-stenotic region [8]. In our study, no patient after CoA repair needed intervention on the aorta during the follow-up period due to dilatation of the ascending aorta or the aortic root.

Concomitant aortic dilatation is seen in 80% of patients with BAV [9]. Studies showed that the dilatation results from a combination of intrinsic aortic wall modifications (genetic theory) and hemodynamic changes induced by the bicuspid valve. The marked heterogenicity of BAV disease leads to different phenotypes, resulting in a large clinical variation of BAV patients [10, 11]. The strong association of BAV with CoA may indicate that BAV disease involves the ascending aorta and aortic arch extending to the ligamentun arteriosum. Dilatation of the ascending aorta occurs as a consequence of aortic medial degeneration [12]. During the follow-up period only one patient with BAV from our study group underwent replacement of the ascending aorta due to aortic aneurysm.

Dilatation of the proximal aorta is a common feature in patients with unrepaired TOF. Corrective surgery has dramatically improved long-term prognosis, and nearly 90% of the patients surviving well into adulthood [13]. However, persistent aortic root dilatation in increasingly reported in adult patients, years after the corrective surgery. In 1997 the first series of progressive aortic root dilatation was published, where a substantial cohort developed subsequent aortic valve incompetence, necessitating reoperation on the aortic root [14].

The underlying mechanism of the aortic dilatation in TOF are both hemodynamic and intrinsic wall abnormalities like cystic medionecrosis as in Marfan syndrome. Presence of right to left shunt shunt, other congenital anomalies, complete repair at older age and even a genetic factor have been implicated in aortic dilatation in TOF [15–17]. In a homogenous cohort of TOF repaired early in infancy, was found that the ascending aortic size decreases with growth of the patient during the first years after surgery, irrespective of the total histology score at surgery [18]. These findings support the presumption that mitigation of the transaortic flow by early surgical repair of TOF triggers a remodeling process that may interrupt the progression of the limited histological alterations of the aortic root, thus preventing late aortic dilatation.

In a recent review by Mongeon et al. [19], in adult patients 35 years after repair of TOF at a mean of 7 years of age, an aortic dimension of ≥40 mm was found in 29% and moderate to severe aortic regurgitation in 3.5% of the patients. Only 3 cases of aortic dissection later after TOF repair have been described, all in severely dilated aortas of ≥70 mm [1].

Moreover, in our study, in the secondary aortopathy group, 50 and 33.30% of the patients had dilated ascending aorta and aortic root respectively.

After the Ross procedure, the neoaortic root dilates mainly at the sinus portion and the sinotubular junction, and less at the neo-aortic annulus itself. Dilatation occurs rapidly within the first days after surgery, with a further increase during the first year of follow-up, without causing significant aortic regurgitation in the medium phase term [20]. The postoperative distention of the pulmonary autograft leads to remodeling of the wall with intimal thickening, medial elastin fragmentation, hypertrophic smooth muscle cells and increased medial and adventitial fibrosis [21]. Freedom from autograft reoperation has been reported between 74 and 93% at 10 years and between 65 to 82% at 15 years [1]. In our study only one patient after the Ross procedure needed reoperation due to pulmonary autograft dilatation during the follow-up period.

In patients with TGA, after the arterial switch operation (ASO), the native pulmonary valve and root assume the role of systemic arterial valve and root. Progressive dilatation of the neo-aortic root exceeds somatic growth during a long follow-up period. A dilated neoaortic root is seen in at least 50% of all patients after ASO [22]. Risk factors that are associated with neo-aortic root dilatation after ASO include the presence of a ventricular septal defect, previous pulmonary artery banding, older age and surgical technical factors [1, 22, 23].

Also, adults patients with TGA after the atrial switch operation have a greater incidence of dilatation of both the pulmonary artery and aorta [24].

Freedom from aortic root reoperation was reported to be between 83 and 97% after ASO [1], Moreover, only a single case report of surgical repair of aneurysm of the ascending aorta after atrial switch operation has been described [25]. In our study group, no patient after ASO or atrial switch operation needed reoperation on the ascending aorta.

Evidence of aortic dilatation has been reported in patients after the Fontan procedure. In a study with a median follow-up of 9 years neo-aortic root dilatation was observed in 98% of the patients [26]. Histological analysis demonstrated findings seen in other forms of CHD- associated aortopathies, such as fragmentation of elastic fibers and deposition of myxoid material [27]. Aortic dissection in patients after the Fontan procedure was reported in two patients with dilated aortic root [1]. In our study group, no patient after the Fontan procedure needed reoperation on the ascending aorta or the aortic root.

One of the main finding of our study is that patients with secondary aortopathy had larger ascending aorta and aortic root dimensions than the patients with primary aortopathy. In the existing literature, so far there are no studies with direct comparison of aortic dimensions between the primary and secondary aortopathy. In our opinion, secondary aortopathy in contrary to the primary aortopathy, the neo-aorta consists mainly of pulmonary artery tissue introduced in the high pressure left- sided system, often leading to more severe dilatation in a time-dependent fashion than in the primary aortopathy.

Regarding aortopathy-associated CHD other than BAV, dissection risk is low [28]. Since, there are no specific guidelines recommendations [3], and no reports suggesting high risk of aortic complications at neo-aortic root/ascending aortic diameters < 55 mm, there is a pragmatic attitude to these patients with a restrictive policy following general aortic disease guidelines (55 mm) [3, 29]. On the other hand, because the time factor is the principal determinant of late neo-aorta dilatation and some cases of quickly progressive diameter increase or fatal complications have been described, a close observation is warranted [3, 30]. In these patients, aortic surgery was performed at lower aortic diameters, particularly if surgery was indicated for aortic valve dysfunction [28].

Regarding BAV the life risk of aortic dissection has been reported to be 9 times higher than that of the general population [1]. Moreover, Kuijpers JM et al. [28], reported 10-year dissection incidence of 0.3%. This low aortic-dissection risk in BAV is also reported in population-based and post- AVR BAV cohorts [28]. Current guideline recommended aortic diameters thresholds for prophylactic surgery in BAV is 55 mm or 50 mm with risk factors, and 45 mm at the time of AVR for dysfunctional BAV [28]. Surgical treatment for ascending aortic dilatation in CoA may be considered when the diameter is > 55 mm (> 27 mm/m^2^) or if rapid progression [31]. Moreover, the close association between BAV and CoA imply strategies established for BAV may be appropriate [3].

In conclusion, progressive aortic root and/or ascending aorta dilatation is frequently found in adults with repaired or unrepaired CHD. Primary aortopathy is associated with BAV, CoA and conotruncal abnormalities, where secondary aortopathy is after congenital heart surgery, by which the original aotic root/ ascending aorta is replaced by a pulmonary autograft, as in Ross procedure or modified as in ASO of Fontan procedure. It was observed that, more patients with secondary aortopathy had dilated ascending aorta and aortic root, as well as larger aortic diameters compare to the patients with primary aortopathy. Routine follow-up of these patients with attention to aortic diameter is necessary.

## Conclusions

The incidence of primary and secondary aortopathy in adults with CHD in our group of patients is 27,56% and 9,45% respectively. More patients with secondary aortopathy had dilated ascending aorta/ aortic root, as well as larger aortic diameters compare to the patients with primary aortopathy. Routine follow-up of these patients with attention to aortic diameter is necessary.

## Data Availability

The datasets used and/or analysed during the current study are available from the corresponding author on request.

## Abbreviations

CHD: Congenital heart disease
CoA: Coarctation of the aorta
BAV: Bicuspid aortic valve
TOF: Tetralogy of Fallot
PA: Pulmonary atresia
VSD: Ventricular septal defect
TAC: Truncus arteriosus
SV: Single ventricle
TTE: Transthoracic echocardiography
TGA: Transposition of great arteries
AVR: Aortic valve replacement
